# Long-Term Outcomes of Endovascular Treatment for Type B Aortic Dissection with Simple Renal Cysts: A Multicenter Retrospective Study

**DOI:** 10.31083/j.rcm2307226

**Published:** 2022-06-24

**Authors:** Hongqiao Zhu, Kaiwen Zhao, Guangkuo Wang, Junjun Liu, Yifei Pei, Jian Zhou, Zaiping Jing

**Affiliations:** ^1^Department of Vascular Surgery, the First Affiliated Hospital of the Navy Medical University, 200433 Shanghai, China; ^2^Department of Vascular Surgery, Jiangmen Central Hospital, 529020 Jiangmen, Guangdong, China; ^3^Department of Vascular Surgery, the Affiliated Hospital of Qingdao University, 266042 Qingdao, Shandong, China

**Keywords:** type B aortic dissection, simple renal cysts, hypertension, thoracic endovascular aortic repair

## Abstract

**Background:**

Few studies have investigated the characteristics and long-term outcomes of type B aortic dissection (BAD) patients with simple renal cysts (SRC) after thoracic endovascular aortic repair (TEVAR).

**Methods:**

A multi-center retrospective cohort study was performed, including 718 BAD patients undergoing TEVAR from 2003 to 2016. The prevalence of SRC was 34.5% (n = 248). After propensity score matching, 214 matched pairs were selected for further analysis. Primary outcomes were long-term aortic-related adverse events (ARAEs). The effects of SRC in each subgroup of interest and their interactions were analyzed.

**Results:**

BAD patients with SRC were older and had a greater prevalence of comorbidities, including hypertension, coronary artery disease and chronic occlusive pulmonary disease. In addition, the SRC group presented a greater proportion of pleural effusion and aortic calcification. Compared with the non-SRC group, a significantly higher maximal diameter of ascending aorta was observed in the SRC group. Apart from the timing of the operation, no differences were found in the medication regime or intra-operative parameters. In the matched population, patients with SRC were at a higher risk of ARAEs in the long term. The multivariable Cox model indicated that SRC was an independent predictor of long-term ARAEs (hazard ratio: 1.84, 95% confidence interval: 1.13–3.00). The interaction between SRC and hypertension on rupture after TEVAR was statistically significant (*p* = 0.023).

**Conclusions:**

Compared with the non-SRC group, BAD patients with SRC experienced a higher risk of long-term ARAEs after TEVAR. Among the SRC subgroup, hypertensive patients had the highest risk of rupture after TEVAR.

## 1. Introduction

Aortic dissection (AD) is a devastating aortic disease caused by an entry tear in the aortic intima or hemorrhage in the aortic media, leading to the separation of the aortic layers [1]. According to the Stanford classification, type B aortic dissection (BAD) originates distal to the ostium of the left subclavian artery [1]. With the development of thoracic endovascular aortic repair (TEVAR), the mortality rate of BAD patients has significantly reduced [2]. Nonetheless, patients can still suffer from diverse stent-graft-related complications, including endoleak, aortic dilation, retrograde type A aortic dissection (AAD) and new dissection [2]. Therefore, it is imperative to identify potential risk factors associated with aortic-related adverse events (ARAEs) in BAD patients undergoing TEVAR.

Recently published studies have found an association between simple renal cysts (SRC) and aortic dissection/aneurysm [3, 4]. However, the perioperative characteristics and long-term outcomes of BAD patients with SRC that undergo TEVAR remain unclear. In this context, a multi-center retrospective study was conducted. First of all, differences in perioperative characteristics and intraoperative details between SRC and non-SRC groups were compared. Moreover, a propensity score matching was performed to minimize selection bias. The differences in long-term outcomes between the two groups were compared in the overall and matched study populations. Finally, subgroup analysis was performed to identify which subpopulations of SRC patients sustained the highest risk of ARAEs after TEVAR.

## 2. Methods

### 2.1 Study Population

**Fig. 1.**
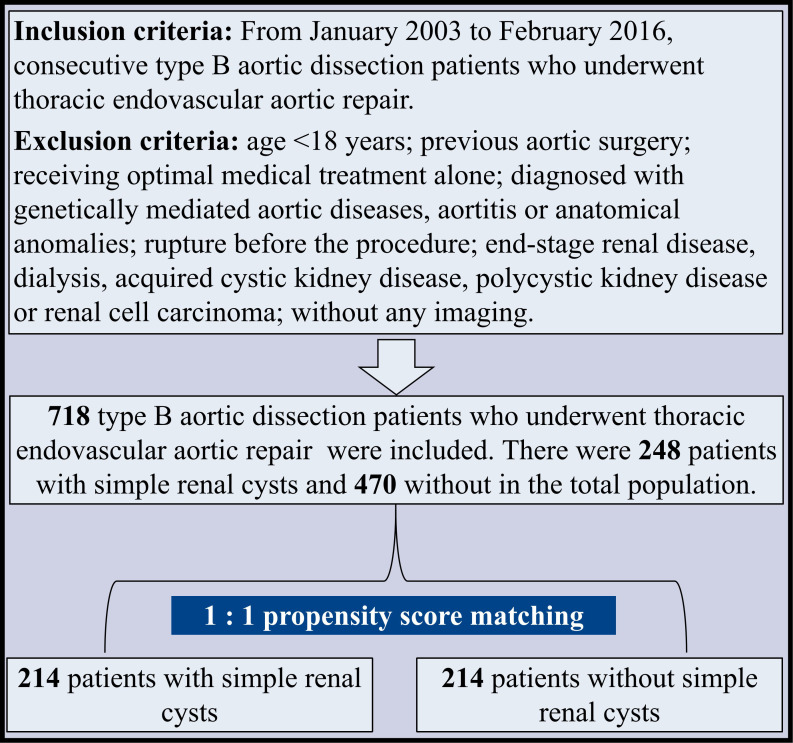
**Flowchart of the study population**. A multi-center retrospective cohort study was performed, including 718 BAD patients undergoing TEVAR from 2003 to 2016, consisting of 248 and 470 patients with and without SRC. After propensity score matching, 214 matched pairs were taken into further analysis.

A multi-center retrospective analysis was performed, including three Chinese tertiary referral centers (**Supplementary Table 1**). Consecutive BAD patients who underwent TEVAR from January 2003 to February 2016 were included in this study. A flowchart of the study population is shown in Fig. 1.

### 2.2 TEVAR Procedure

The indications of TEVAR for complicated and uncomplicated BAD were reported in our previous studies [5]. All procedures were performed as previously described [5, 6].

### 2.3 Study Definitions and Follow-Up

The duration of BAD was divided into acute (<15 days after onset), subacute (15–90 days) and chronic phases (>90 days) [7]. CTA data on the admission of the eligible patients were retrospectively reviewed to assess the anatomical characteristics, including arch type, aortic calcification, thrombosis status of the false lumen, maximal diameter of the ascending and descending aorta according to the guidelines of the Society for Vascular Surgery [8].

The presence of SRC was confirmed by the CT or magnetic resonance imaging on admission. The same definition was used to diagnose SRC in all participating centers: a lesion characterized with round shape, thin wall, size ≥4 mm, with low attenuation and no distinct enhancement or septations radiographically [9]. BAD patients were then divided into SRC and non-SRC groups.

Primary outcomes were defined as any ARAEs, including endoleak (type I, II and III), new dissection, retrograde AAD, aortic dilation, rupture (of the false lumen) and aortic-related mortality at 5-year follow-up [2]. The in-hospital aortic-related mortality was evaluated by reviewing the inpatient record, death and autopsy reports, while the out-of-hospital mortality was determined by phone calls. Secondary outcomes included all-cause mortality and cardiovascular events at the five-year follow-up. A short-term outcome was defined as any clinical adverse event described at 30-day follow-up, while a long-term outcome was defined as any events occurring at the 5-year follow-up. The follow-up was performed until February 2021. The completeness of follow-up of our study was estimated using the Clark C index [10].

### 2.4 Statistical Analysis

Data were presented as n (%) for categorical variables and mean ± standard deviations or median (interquartile range, IQR) for continuous variables. The Chi-square test or Fisher exact test was used to compare categorical variables, while the student *t*-test was used to compare continuous variables.

To minimize selection bias and improve confounding variable balance, adverse clinical outcomes were compared using propensity-matched data for the 214 pairs of SRC and non-SRC patients. The propensity score matching was conducted using a caliper width of ±0.1. Maximal SMD was usually considered acceptable with a value <0.2 [11, 12]. All variables in Tables 1,2 were included in the matching process.

**Table 1.** **Preoperative characteristics of patients with and without SRC and propensity-matched population**.

Variables		Overall				Matched population			
		SRC group	Non-SRC group	SMD	*p* value	SRC group	Non-SRC group	SMD	*p* value
		n = 248	n = 470			n = 214	n = 214		
Baseline characteristics									
	Age (y)	61.1 ± 12.6	56.3 ± 12.7	0.38	<0.001	59.5 ± 12.4	60.3 ± 13.8	0.06	0.494
	Male	199 (80.2%)	396 (84.3%)	0.11	0.175	172 (80.4%)	176 (82.2%)	0.05	0.62
	BMI	24.4 ± 3.6	24.7 ± 3.6	0.1	0.254	24.6 ± 3.7	24.6 ± 3.4	0.01	0.893
	Smoking	135 (54.4%)	245 (52.1%)	0.05	0.556	116 (54.2%)	120 (56.1%)	0.04	0.697
	Drinking	47 (19.0%)	99 (21.1%)	0.05	0.504	44 (20.6%)	45 (21.0%)	0.01	0.905
	Hypertension	205 (82.7%)	349 (74.3%)	0.21	0.011	174 (81.31%)	169 (78.97%)	0.06	0.545
	CAD	22 (8.9%)	18 (3.8%)	0.21	0.005	12 (5.6%)	14 (6.5%)	0.04	0.686
	Arrhythmia	32 (12.9%)	52 (11.1%)	0.06	0.466	25 (11.7%)	34 (15.9%)	0.12	0.207
	Stroke	10 (4.03%)	17 (3.62%)	0.02	0.781	8 (3.7%)	11 (5.1%)	0.07	0.481
	COPD	46 (18.6%)	27 (5.7%)	0.40	<0.001	23 (10.8%)	26 (12.2%)	0.04	0.649
	Diabetes mellitus	21 (8.5%)	37 (7.9%)	0.02	0.781	16 (7.5%)	24 (11.2%)	0.13	0.184
	CKD	16 (6.5%)	19 (4.0%)	0.11	0.154	14 (6.5%)	8 (3.7%)	0.13	0.189
	SBP at admission (mmHg)	138.7 ± 21.9	138.0 ± 21.2	0.03	0.669	137.9 ± 21.7	137.6 ± 20.1	0.01	0.956
	DBP at admission (mmHg)	82.9 ± 11.32	82.3 ± 12.0	0.05	0.509	83.0 ± 11.5	81.2 ± 11.5	0.15	0.115
	Pleural effusion	104 (41.9%)	127 (27.0%)	0.32	<0.001	84 (39.3%)	76 (35.5%)	0.08	0.424
	Malperfusion	18 (7.26%)	23 (4.89%)	0.1	0.194	16 (7.5%)	9 (4.2%)	0.14	0.149
Echocardiography parameters									
	Diameters of ascending aorta (cm)	3.4 ± 0.5	3.4 ± 0.6	0.06	0.643	3.4 ± 0.5	3.4 ± 0.6	0.06	0.677
	LVEF (%)	61.8 ± 4.9	62.2 ± 5.1	0.08	0.434	61.9 ± 5.0	62.5± 4.9	0.12	0.482
Medication in hospital									
	ACEI/ARB	140 (56.5%)	239 (50.9%)	0.11	0.153	111 (51.87%)	112 (52.34%)	0.01	0.923
	α-blocker	96 (38.7%)	187 (39.8%)	0.02	0.779	81 (37.9%)	86 (40.2%)	0.05	0.62
	β-blocker	167 (67.3%)	339 (72.1%)	0.1	0.181	143 (66.8%)	146 (68.2%)	0.03	0.757
	CCB	187 (75.4%)	352 (74.9%)	0.01	0.881	160 (74.8%)	147 (68.7%)	0.14	0.163
	Diuretic	33 (13.3%)	60 (12.8%)	0.02	0.838	27 (12.6%)	35 (16.4%)	0.11	0.272
Anatomical characteristics									
Arch type				0.07	0.641			0.12	0.459
	I	83 (33.5%)	157 (33.4%)			68 (31.8%)	71 (33.2%)		
	II	32 (12.9%)	50 (10.6%)			27 (12.6%)	19 (8.9%)		
	III	133 (53.6%)	263 (56.0%)			119 (55.6%)	124 (57.9%)		
Diameters of maximum ascending aorta (mm)		40.2 ± 5.2	38.3 ± 5.4	0.35	0.012	38.9 ± 5.3	38.2 ± 6.3	0.12	0.413
Diameters of maximum descending aorta (mm)		44.1 ± 11.7	44.1 ± 12.4	0.01	0.97	44.3 ± 11.8	44.2 ± 11.1	0.01	0.772
Calcification				0.66	<0.001			0.13	0.595
	None	93 (37.5%)	319 (67.9%)			87 (40.65%)	95 (44.39%)		
	Mild	105 (42.3%)	117 (24.9%)			89 (41.59%)	91 (42.52%)		
	Moderate	40 (16.1%)	26 (5.5%)			31 (14.49%)	23 (10.75%)		
	Severe	10 (4.0%)	8 (1.7%)			7 (3.27%)	5 (2.34%)		
Thrombosis				0.15	0.331			0.08	0.879
	Patent	113 (45.6%)	182 (38.7%)			95 (44.4%)	90 (42.1%)		
	Partial	83 (33.5%)	174 (37.0%)			74 (34.6%)	72 (33.6%)		
	Complete	34 (13.7%)	79 (16.8%)			29 (13.6%)	34 (15.9%)		
	ULP	18 (7.3%)	35 (7.5%)			16 (7.5%)	18 (8.4%)		

Values are reported as n (%), mean ± standard deviations or median (interquartile range).

**Table 2.** **Intra-operative details of patients with and without SRC and propensity-matched population**.

Variables		Overall				Matched population			
		SRC group	Non-SRC group	SMD	*p* value	SRC group	Non-SRC group	SMD	*p* value
		n = 248	n = 470			n = 214	n = 214		
Operation time (min)		120.0 (79.0–156.3)	115.0 (80.0–160.0)	0.07	0.448	120.0 (80.0–160.0)	115.0 (80.0–150.0)	0.07	0.629
Oversize (%)		15.8 (7.4–23.5)	16.1 (7.4–33.5)	0.45	0.074	16.1 (8.0–27.1)	12.5 (6.6–28.7)	0.05	0.792
Length of proximal landing zone (mm)		24.0 (17.3–32.8)	20.6 (14.1–30.3)	0.22	0.239	22.4 (15.0–32.3)	22.0 (15.8–34.8)	0.02	0.802
Timing of operation				0.22	0.02			0.05	0.856
	Acute phase	153 (61.7%)	239 (50.9%)			128 (59.8%)	127 (59.4%)		
	Subacute phase	64 (25.8%)	160 (34.0%)			59 (27.6%)	63 (29.4%)		
	Chronic phase	31 (12.5%)	71 (15.1%)			27 (12.6%)	24 (11.2%)		
Hybrid approach		1 (0.4%)	10 (2.1%)	0.15	0.074	1 (0.5%)	4 (1.9%)	0.13	0.372
Chimney technique		51 (20.6%)	77 (16.4%)	0.11	0.164	45 (21.0%)	36 (16.8%)	0.11	0.267
Adjunctive procedure		44 (17.7%)	97 (20.6%)	0.07	0.353	40 (18.7%)	45 (21.0%)	0.06	0.545
Era				0.03	0.697			0.03	0.771
	2003–2010	106 (42.7%)	208 (44.3%)			98 (45.8%)	95 (44.4%)		
	2011–2016	142 (57.3%)	262 (55.7%)			116 (54.2%)	119 (55.6%)		

Values are reported as n (%) or median (interquartile range).

To identify potential risk factors for ARAEs, Cox hazard analysis was used in the matched population. Variables that had a significant correlation (*p *< 0.1) in the univariate analysis were selected for multivariable analysis using the backward selection method. All tests were two-sided, and a *p*-value < 0.05 was statistically significant. Statistical package R version 3.4.3 (R Foundation for Statistical Computing, Vienna, Austria) was used to analyze the data.

## 3. Results

### 3.1 Clinical Characteristics

From January 2003 to February 2016, 718 patients were enrolled in the study, of whom 595 were males (82.9%), and 248 presented with at least one SRC (34.5%) (Table 1). Patients with SRC were older (61.1 ± 12.6 years versus 56.3 ± 12.7 years; *p *< 0.001) and had a greater prevalence of comorbidities, including hypertension (*p* = 0.011), coronary artery disease (CAD) (*p* = 0.005) as well as chronic occlusive pulmonary disease (COPD) (*p *< 0.001). Compared with the non-SRC group, BAD patients with SRC presented a greater pleural effusion prevalence (*p *< 0.001). Anatomically, a greater proportion of patients with SRC presented with aortic calcification (*p *< 0.001) and the maximal diameter of the ascending aorta was significantly larger in patients with SRC (*p* = 0.012).

### 3.2 Intra-Operative Details

Table 2 shows the differences in intra-operative details between the SRC and non-SRC groups.

### 3.3 Outcomes

Short-term outcomes in the overall and matched population were shown in the **Supplementary Table 2**.

**Fig. 2.**
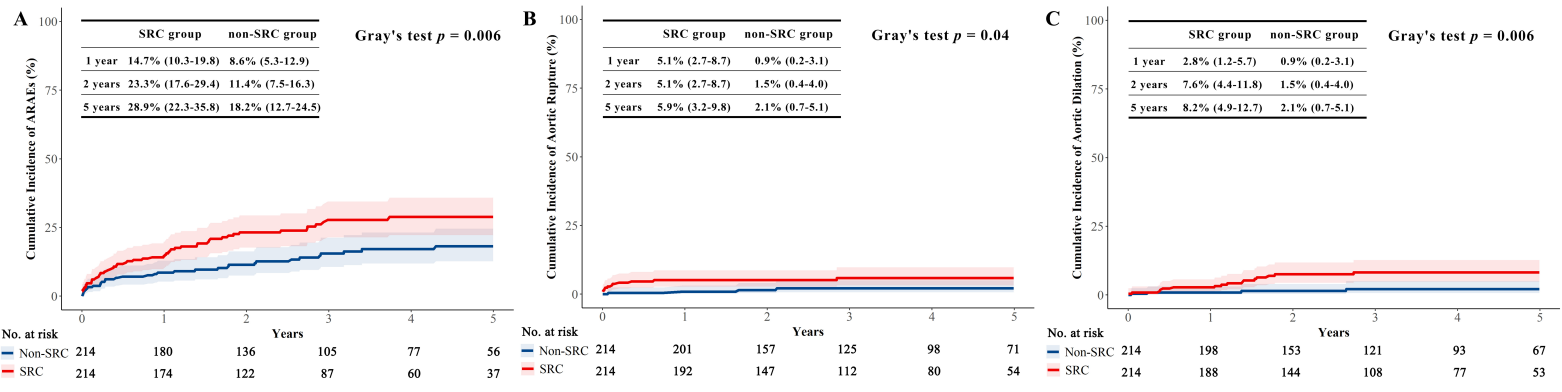
**The cumulative incidences of the ARAEs, rupture and aortic dilation, with all-cause death as the competing risk in the matched population**. (A)The cumulative incidence of ARAEs in the SRC group was significantly greater than in the non-SRC group (*p* = 0.006). (B) The cumulative incidence of rupture in the SRC group was significantly greater than in the non-SRC group (*p* = 0.04). (C) The cumulative incidence of aortic dilation in the SRC group was significantly greater than in the non-SRC group (*p* = 0.006). The differences were assessed with Gray’s test.

**Fig. 3.**
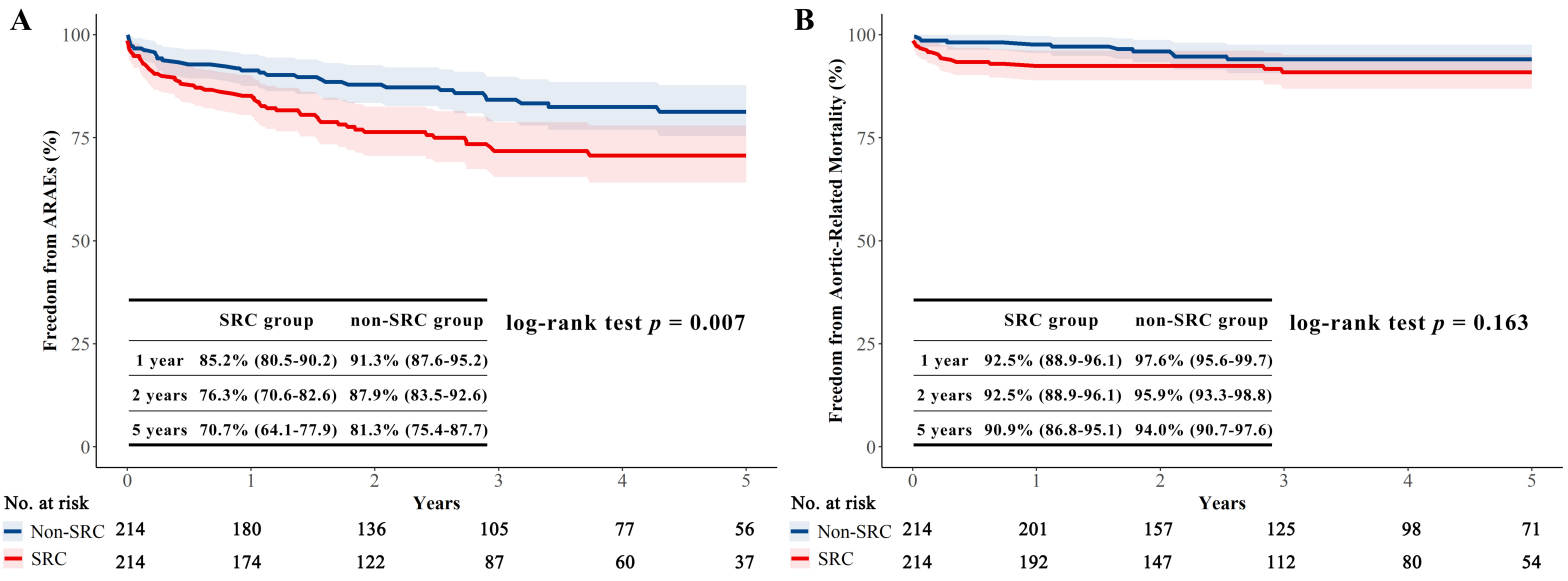
**Kaplan-Meier survival analysis of ARAEs and aortic-related mortality in the matched population**. (A) Freedom from ARAEs between the SRC and non-SRC groups. Freedom from ARAEs in the SRC group was significantly lower than that of the non-SRC group (*p* = 0.007). (B) Freedom from aortic-related mortality between the two groups. Freedom from aortic-related mortality in the SRC group was similar with that of the non-SRC group (*p* = 0.163). The differences between the SRC and non-SRC groups were assessed with log-rank test.

The median length of follow-up in the matched population was 3.6 (IQR: 1.7–5.9) years and 3.1 (IQR: 1.2–5.7) years for patients with and without SRC, respectively. The completeness of follow-up for the primary outcomes was 67.3% for the SRC group and 68.1% for the non-SRC group. In the matched population, ARAEs were significantly lower in the non-SRC group than in the SRC group (Gray’s test *p* = 0.006 and log-rank *p* = 0.007) (Fig. 2A and Fig. 3A). In terms of 5-year aortic rupture, the cumulative incidence was 5.9% in the SRC group and 2.1% in the non-SRC group (*p* = 0.04) (Fig. 2B). Cumulative incidence of 5-year aortic dilation was also higher in patients with SRC (8.2% vs. 2.1%, *p* = 0.006) (Fig. 2C). No difference was observed in the aortic-related mortality between the two groups (log-rank *p* = 0.163) (Fig. 3B). **Supplementary Figs. 1–4** show the cumulative incidences of aortic-related mortality, endoleak, retrograde AAD and new dissection in the matched population. Long-term outcomes in the total population are presented in the **Supplementary Table 3**.

### 3.4 Outcomes Analysis

Univariate Cox hazard analysis indicated that SRC, hypertension, chronic occlusive pulmonary disease, operation time, stroke, malperfusion, chronic aortic dissection, maximum diameters of descending aorta were potential risk factors for ARAEs (*p *< 0.1) (**Supplementary Table 4**). Multivariable Cox hazard analysis revealed that SRC (HR: 1.84, 95% CI: 1.13–3.00), stroke (HR: 2.62, 95% CI: 1.31–5.24), chronic aortic dissection (HR: 2.16, 95% CI: 1.16–4.02) were independent risk factors of ARAEs (Table 3).

**Table 3.** **Multivariable Cox hazard analysis of ARAEs in the matched population**.

Variables		Hazard ratio (95% CI)	*p* value
SRC		1.84 (1.13, 3.00)	0.014
Stroke		2.62 (1.31, 5.24)	0.006
Timing of operation			
	Acute phase	Reference	
	Subacute phase	1.56 (0.92, 2.64)	0.098
	Chronic phase	2.16 (1.16, 4.02)	0.015

As seen in Table 4, a statistically significant interaction between SRC and hypertension on the risk of rupture after TEVAR was found (*p* = 0.023).

**Table 4.** **Multivariable Cox analyses in the matched population for ARAEs, aortic-related mortality, rupture, endoleak, aortic dilation, new dissection and retrograde AAD**.

Outcomes		Hazard ratio (95% CI)	*p* value	*p* for interaction*
ARAEs				0.917
	Overall	1.84 (1.13–3.00)	0.014	
	Normotension	1.64 (0.55–4.89)	0.372	
	Hypertension	1.77 (1.09–2.85)	0.02	
Aortic-related mortality				0.472
	Overall	1.74 (0.82–3.69)	0.147	
	Normotension	0.7 (0.06–7.75)	0.773	
	Hypertension	1.78 (0.79–4.00)	0.161	
Rupture				0.023
	Overall	3.13 (1.01–9.72)	0.048	
	Normotension	0.92 (0.15–5.51)	0.926	
	Hypertension	5.58 (1.25–24.95)	0.024	
Endoleak				0.398
	Overall	1.19 (0.43–3.28)	0.739	
	Normotension	2.81 (0.26–31.0)	0.399	
	Hypertension	0.96 (0.31–2.98)	0.946	
Aortic dilation				0.918
	Overall	4.17 (1.39–12.48)	0.011	
	Normotension	4.05 (0.42–38.98)	0.226	
	Hypertension	4.27 (1.21–15.00)	0.023	
New dissection				NA
	Overall	1.11 (0.07–17.8)	0.942	
	Normotension	NA	NA	
	Hypertension	1.07 (0.07–17.21)	0.961	
Retrograde AD				0.602
	Overall	1.29 (0.53–3.12)	0.573	
	Normotension	0.73 (0.07–8.01)	0.794	
	Hypertension	1.39 (0.53–3.64)	0.509	

*Interactions between SRC and hypertension on outcomes were investigated.

When patients were stratified according to potential impact factors for ARAEs, no significant interactions with SRC were found for age, hypertension, stroke, operation timing, chimney technique, adjunctive procedure and hybrid approach (**Supplementary Table 5**).

## 4. Comment

Although previous studies indicated that SRC is a significant risk factor for BAD patients undergoing TEVAR [4], the relatively short follow-up periods and small sample size in these studies could be a source of selection and observational bias. To our best knowledge, this is the largest and most contemporary report providing comprehensive analyses of SRC-related disparities in baseline characteristics, anatomical patterns, intra-operative details and long-term outcomes of BAD patients after TEVAR.

In our study, BAD patients with SRC were significantly older than those without, consistent with the literature [13]. In comparison with the general population, patients with BAD were found to be older [14]. Clinical evidence from the international registry of acute aortic dissection (IRAD) showed that the mean age of BAD patients was 63.5 years (the mean age in our study is 57.9 years) [15]. Moreover, we found that the SRC group was significantly older than the non-SRC group (mean age, 61.1 years vs. 56.3 years; *p *< 0.001). However, no significant interaction between age and SRC on ARAEs was found (*p* for interaction = 0.429, **Supplementary Table 5**). Our results indicated that age, although related to SRC, is not the major factor influencing aortic-related outcomes in BAD patients after TEVAR.

A significant gender imbalance was found in SRC patients in our study, with a male-to-female ratio of 4.8:1. Moreover, female patients were significantly older than male patients (62.1 ± 12.9 years vs. 57.1 ± 12.7 years, *p *< 0.001), while the prevalence of SRC between the two groups was similar (39.8% vs. 33.4%, *p* = 0.175). In a study by Chung *et al*. [16], female patients that underwent cardiac surgery were older with larger indexed aortic sizes, with a lower prevalence of coronary disease and reduced left ventricular ejection fraction than their male counterparts. In our study, the prevalence of CAD (*p* = 0.174), stroke (*p* = 0.781), COPD (*p* = 0.07) and CKD (*p* = 0.357) was similar between the males and females, while hypertension was predominantly found in male patients (*p *< 0.001).

In our study, a difference was found in the prevalence of hypertension between the two groups in the total population (Table 1). Consistently, previous clinical studies have shown that SRC is associated with a greater prevalence of hypertension [17]. Although we found that hypertension is not an independent predictor of ARAEs, the subgroup analysis showed that patients with SRC and hypertension led to the highest risk of rupture after TEVAR (Table 4). The attenuation of the power to detect a direct association between hypertension and ARAEs may also result from relatively small sample (n = 214) after matching. Recently, Lu *et al*. [4] found that lower diastolic blood pressure at admission could predict ARAEs in BAD patients after TEVAR, which emphasized the important role of hemodynamic stability in aortic remodeling. Whether hypertensive patients with SRC suffer from severer blood pressure fluctuation than that without after onset of BAD warrants further study.

Contrary to the previous belief that SRC is a benign disease, an increasing body of evidence suggests that patients with SRC experience a higher risk of long-term ARAEs after TEVAR [3, 4]. What’s more, no difference in 5-year all-cause mortality or cardiovascular events (shown in **Supplementary Figs. 5,6**) was found between the two groups, implying that SRC might play an exclusive role in the adverse aortic remodeling after TEVAR.

We noticed that the difference of ARAEs between the two groups was mostly accumulated in the first 2 years (Fig. 2). Therefore, a landmark analysis was performed by dividing the 5-year follow-up into the first two years and the remaining three years. Results indicated that during the first two years, patients with SRC were at a higher risk of ARAEs (HR: 1.93, 95% CI: 1.17–3.19, *p* = 0.009). However, for the remaining three years, the cumulative hazard ratios in the two groups were similar (HR: 1.02, 95% CI: 0.37–2.82, *p* = 0.97). One explanation for this trend is that the patients initially without SRC could have developed new cysts, increasing the probability of ARAEs at follow-up. Another explanation is that all patients receiving TEVAR could benefit from positive aortic remodeling in the long term, irrespective of the presence of SRC [18]. In any case, further studies should be conducted to identify the role of SRC on long-term patient outcomes.

According to current clinical and laboratory evidence, there are several possible underlying correlations between SRC and BAD.

First, both SRC and BAD are related to increased matrix metalloproteinases (MMPs). It has been established that the structural integrity of the aorta depends on the expression of extracellular matrix (ECM) proteins, which are regulated by proteolytic enzymes. Zhang *et al*. [19] found that MMP-1, MMP-9, and active MMP-9 levels were higher in aortic dissection tissue than control tissue. Interestingly, MMP overexpression and an elevated ratio of MMPs to tissue inhibitors of metalloproteinases (TIMPs) in aortic tissue could induce the degradation of multiple components in the ECM, making the blood vessel more vulnerable to adverse clinical events [20]. Similarly, Harada *et al*. [21] found massive accumulation of MMP-2 and MMP-9 in human benign cystic fluids. Obermuller *et al*. [22] found the upregulation of MMP-14 in a rat model of autosomal-dominant polycystic kidney disease and advocated that TIMPs are promising biomarkers for treating polycystic kidney. In summary, the imbalance of MMPs and TIMPs may persist in patients with SRC and BAD, making them susceptible to ARAEs after TEVAR.

Moreover, our study indicated that the patients with SRC had a greater aortic calcification prevalence than the non-SRC group in the total population. It has been established that multiple factors can affect arterial calcification, including calcium and phosphorus imbalances [23]. Calcium ions are key mediators that regulate inflammation, apoptosis and calcification in vascular smooth muscle cells (VSMC) [24]. Consistently, decreased calcium concentration has been documented in AD patients [25], which could be attributed to calcium loss resulting from the administration of diuretics for blood pressure control [26]. Interestingly, clinical studies demonstrated that ${\mathrm{Ca}}^{2+}$-antagonists were related to the enlargement of simple renal cysts [27]. Clinically, calcification-induced degradation presents a reduction in stiffness of the aortic wall [23]. This degradation, coupled with the significant tension caused by calcification, results in a remarkable increase in stress around the non-calcified aortic tissue, ultimately putting the aorta at high risk of rupture. Disorders of calcium metabolism and aortic calcification may lead to chronic inflammation of the arterial wall and the formation of residual thrombosis [28], consistent with our findings.

Overall, the results of our study suggested that SRC may be an independent high-risk predictor of long-term ARAEs in BAD patients after TEVAR. According to our findings, BAD patients with SRC should be followed-up by CTA at 1-month, 1-year and 2-year follow-ups and blood pressure should be closely monitored within two years after TEVAR. Annual CTA follow-up and routine blood pressure monitor should be recommended in patients with SRC, two years after TEVAR.

## 5. Limitation

Due to the retrospective design of our study, it was impossible to randomly allocate the patients.

## 6. Conclusions

Compared with the non-SRC group, BAD patients with SRC experienced a higher risk of long-term ARAEs after TEVAR. Subgroup analysis showed that the presence of SRC and hypertension led to the highest risk of rupture after TEVAR.

## Data Availability

The datasets used and/or analyzed during the current study are available from the corresponding author on reasonable request.

## Abbreviations

AD, aortic dissection; BAD, type B aortic dissection; SRC, simple renal cysts; TEVAR, thoracic endovascular aortic repair; AAD, type A aortic dissection; Retrograde AAD, Retrograde type A aortic dissection; ARAEs, aortic-related adverse events; PSM, propensity score matching; CAD, coronary artery disease; COPD, chronic occlusive pulmonary disease; CKD, chronic kidney disease; MMPs, matrix metalloproteinases; CTA, computed tomographic angiogram; DSA, digital subtraction angiography; IQR, interquartile range; SMD, standardized mean difference; HR, hazard ratio; CI, confidence interval; LVEF, left ventricular ejection fraction; IRAD, international registry of acute aortic dissection; ECM, extracellular matrix; TIMPs, tissue inhibitors of metalloproteinases; VSMC, vascular smooth muscle cells; BMI, body mass index; SBP, systolic blood pressure; DBP, diastolic blood pressure; ACEI/ARB, angiotensin-converting enzyme inhibitor/angiotensin receptor blocker; ULP, ulcer-like projection.

## Supplementary Material

Supplementary material associated with this article can be found, in the online version, at https://doi.org/10.31083/j.rcm2307226.
