# Supplementary material for: Long-Term Outcomes of Endovascular Treatment for Type B Aortic Dissection with Simple Renal Cysts: A Multicenter Retrospective Study
Source: Rev Cardiovasc Med. 2022 Jun 24;23(7):226. doi: 10.31083/j.rcm2307226 (PMC11266766; doi:10.31083/j.rcm2307226)
Supplement: Supplementary file 1 [file 2153-8174-23-7-226-s1.zip › 2153-8174-23-7-226-s1/v0-Supplementary Figures.docx]

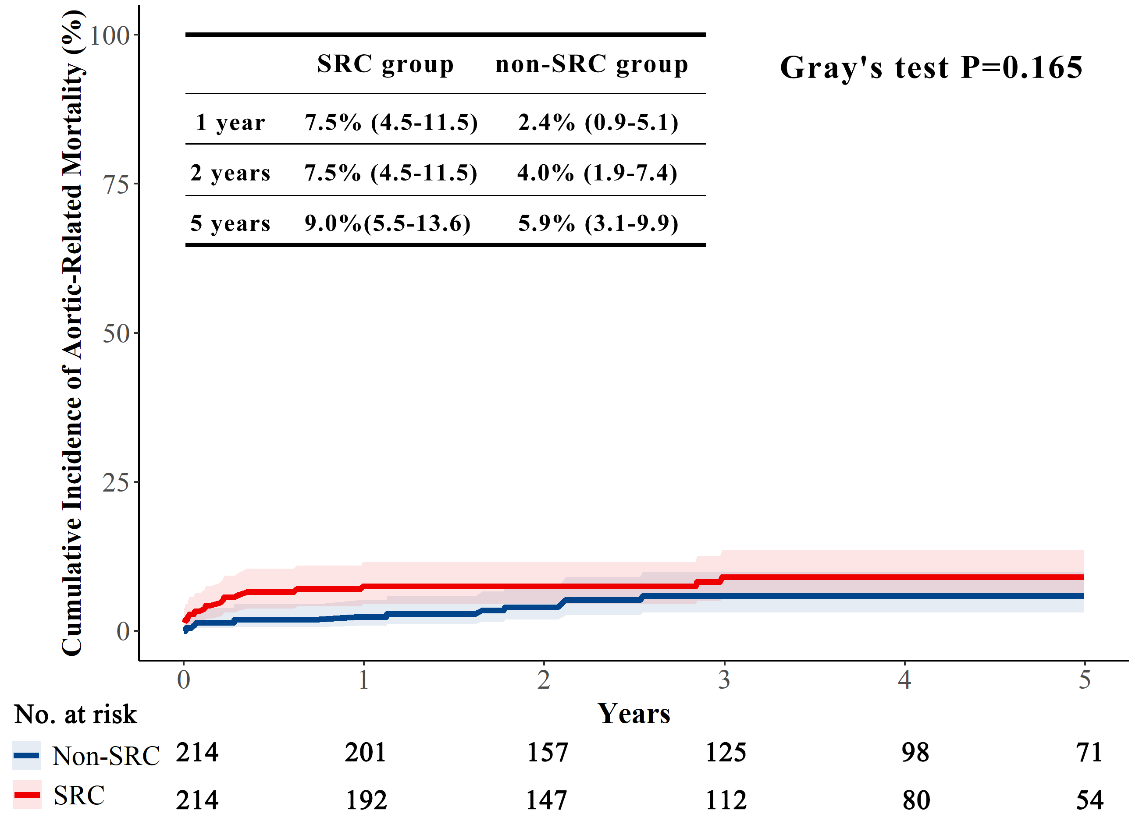


**Supplementary Fig. 1. The cumulative incidence of aortic-related mortality with nonaorta-related death as a competing risk in the matched population.** The differences were assessed with Gray's test. The cumulative incidence of aortic-related mortality between the two groups was similar (*p* = 0.165). SRC, simple renal cysts.


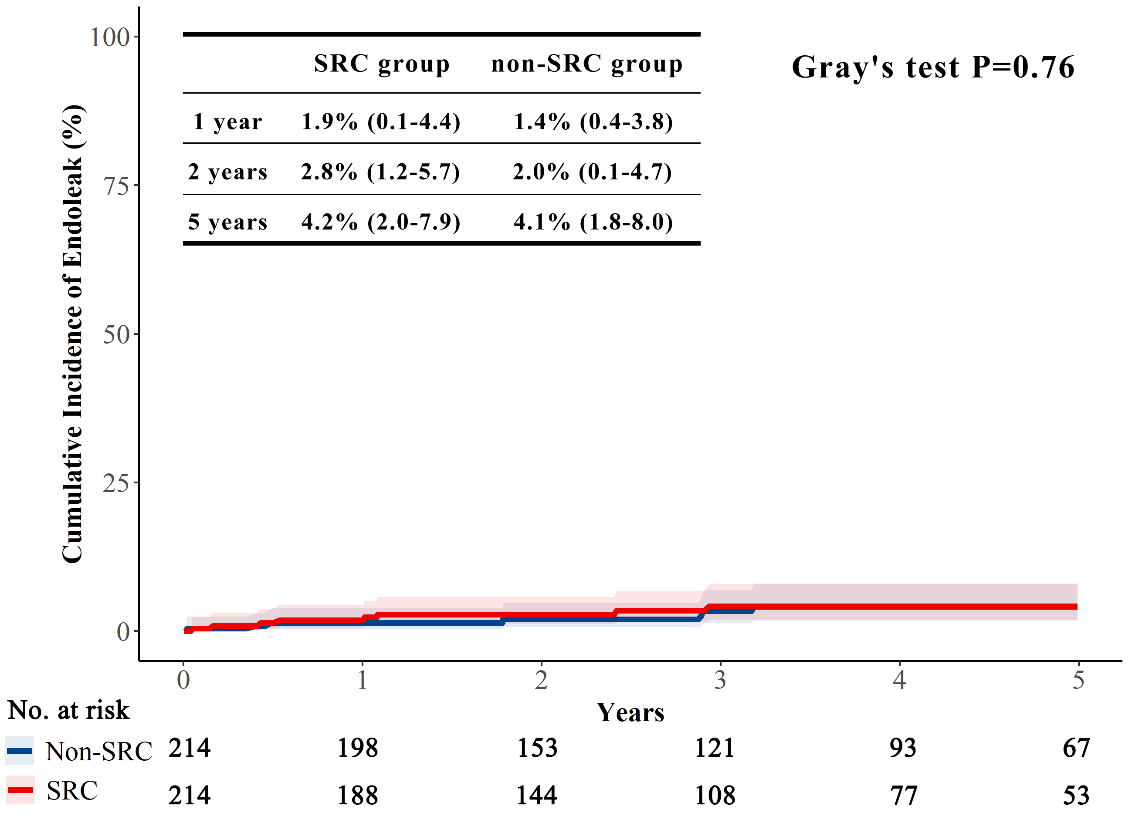


**Supplementary Fig. 2. The cumulative incidence of the endoleak with all-cause death as a competing risk in the matched population.** The differences were assessed with Gray's test. The cumulative incidence of endoleak between the two groups was similar (*p* = 0.76). SRC, simple renal cysts.


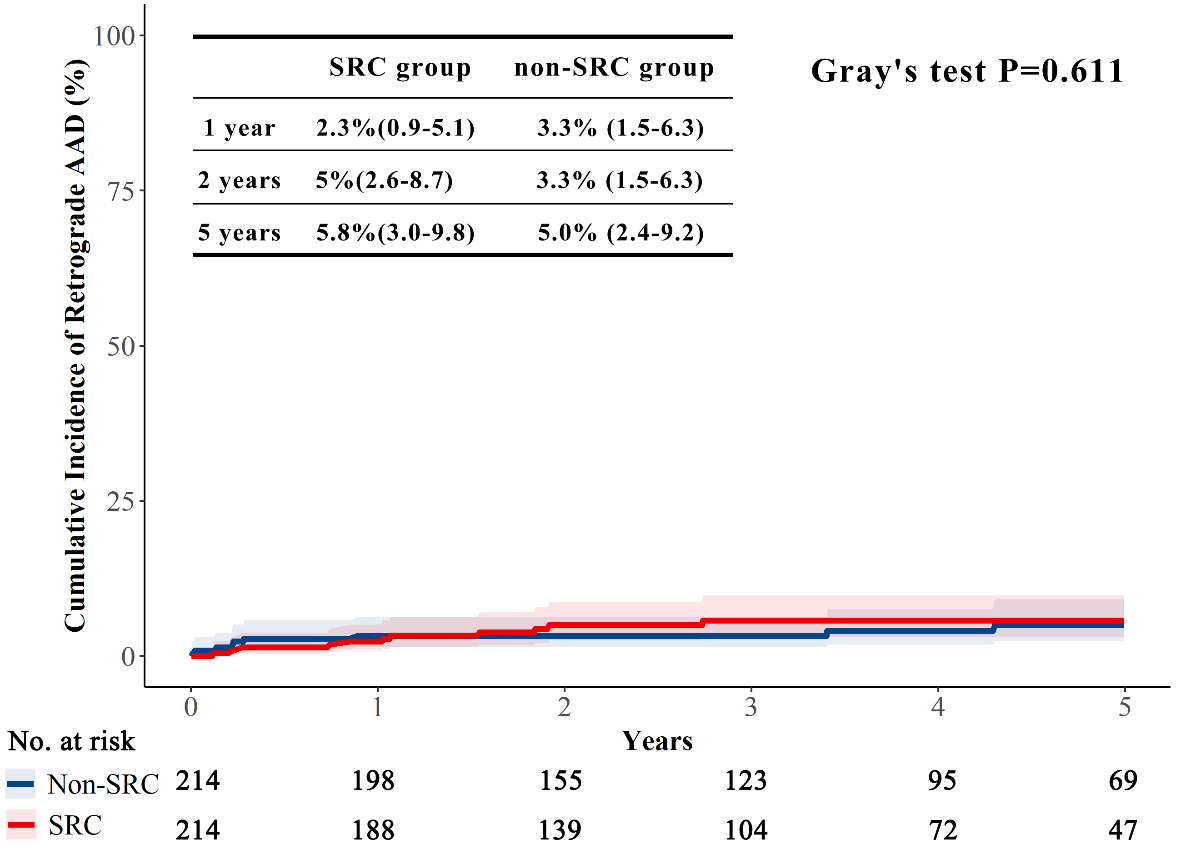


**Supplementary Fig. 3. The cumulative incidence of the retrograde AAD with all-cause death as a competing risk in the matched population.** The differences were assessed with Gray's test. The cumulative incidence of retrograde AAD between the two groups was similar (*p* = 0.611). AAD, type A aortic dissection; SRC, simple renal cysts.


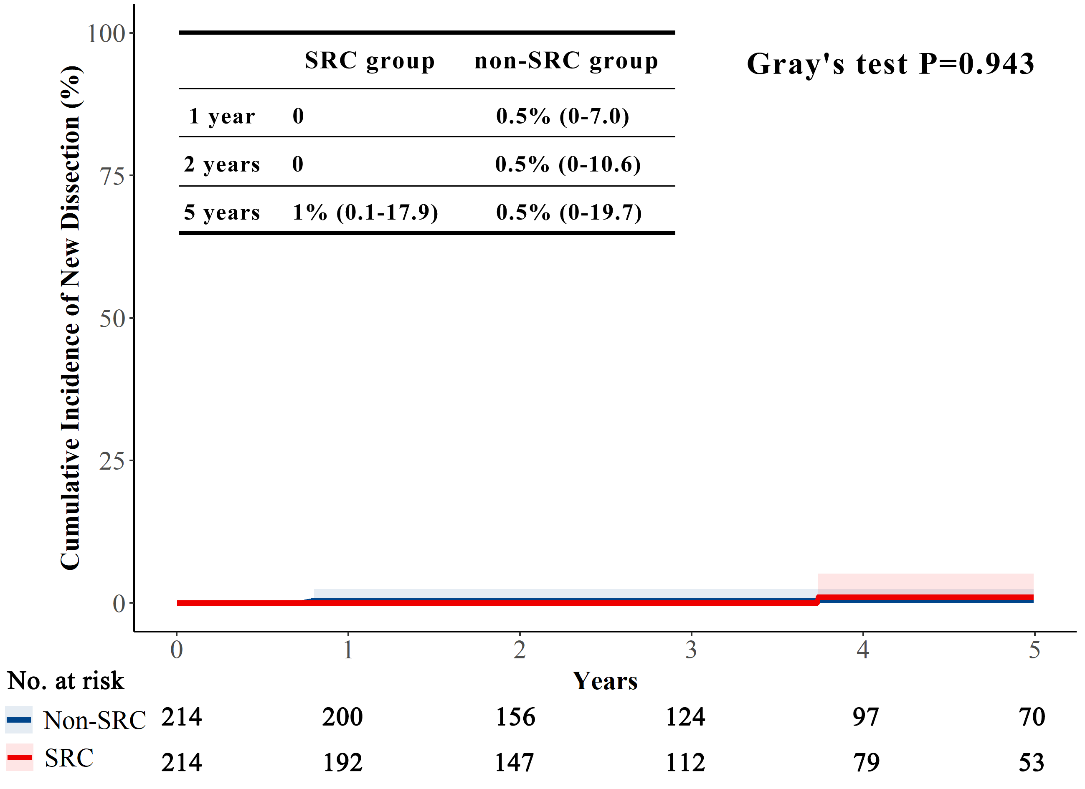


**Supplementary Fig. 4.** **The cumulative incidence of the new dissection with nonaorta-related death as a competing risk in the matched population.** The differences were assessed with Gray's test. The cumulative incidence of aortic-related mortality between the two groups was similar (*p* = 0.943). SRC, simple renal cysts.
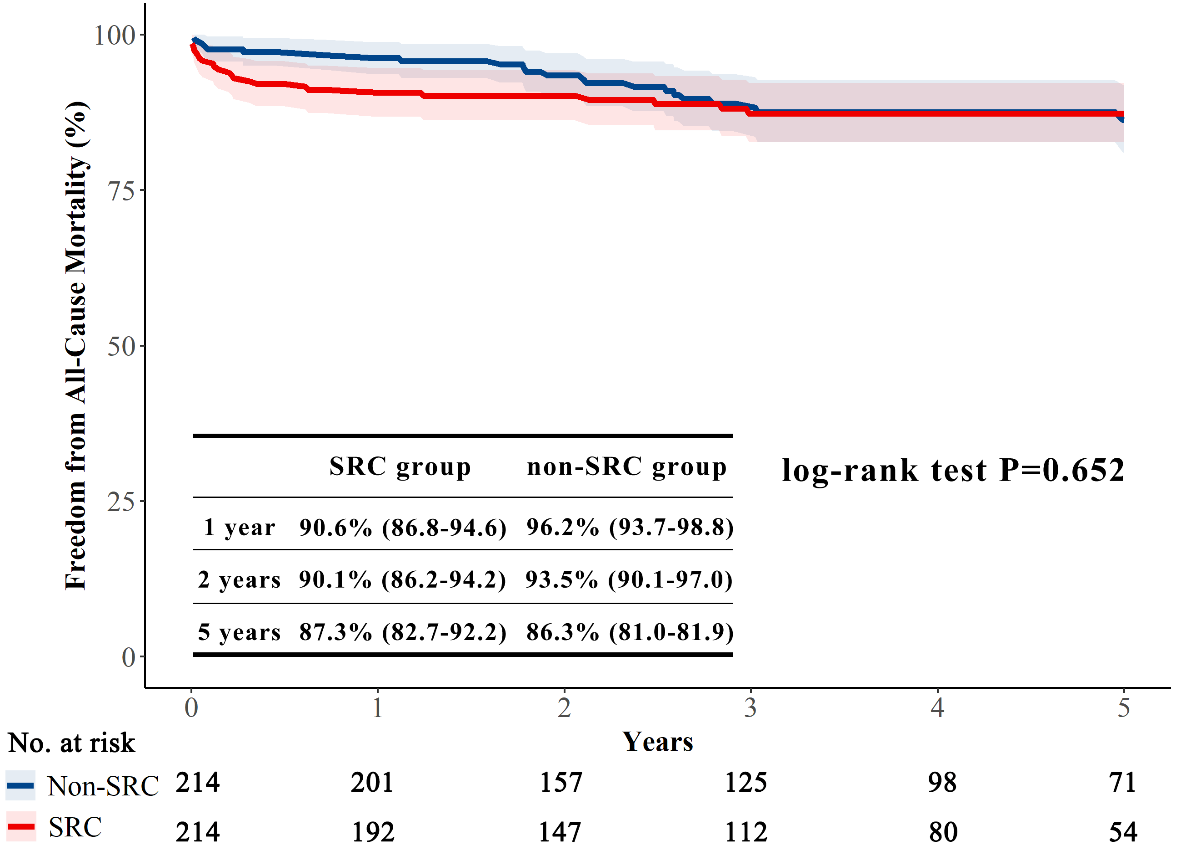


**Supplementary Fig. 5. Kaplan-Meier survival analysis of all-cause mortality in the matched population.** The difference between the SRC and non-SRC groups was assessed with the log-rank test. Freedom from all-cause mortality in the SRC group was similar to that of the non-SRC group (*p* = 0.652). SRC, simple renal cysts.


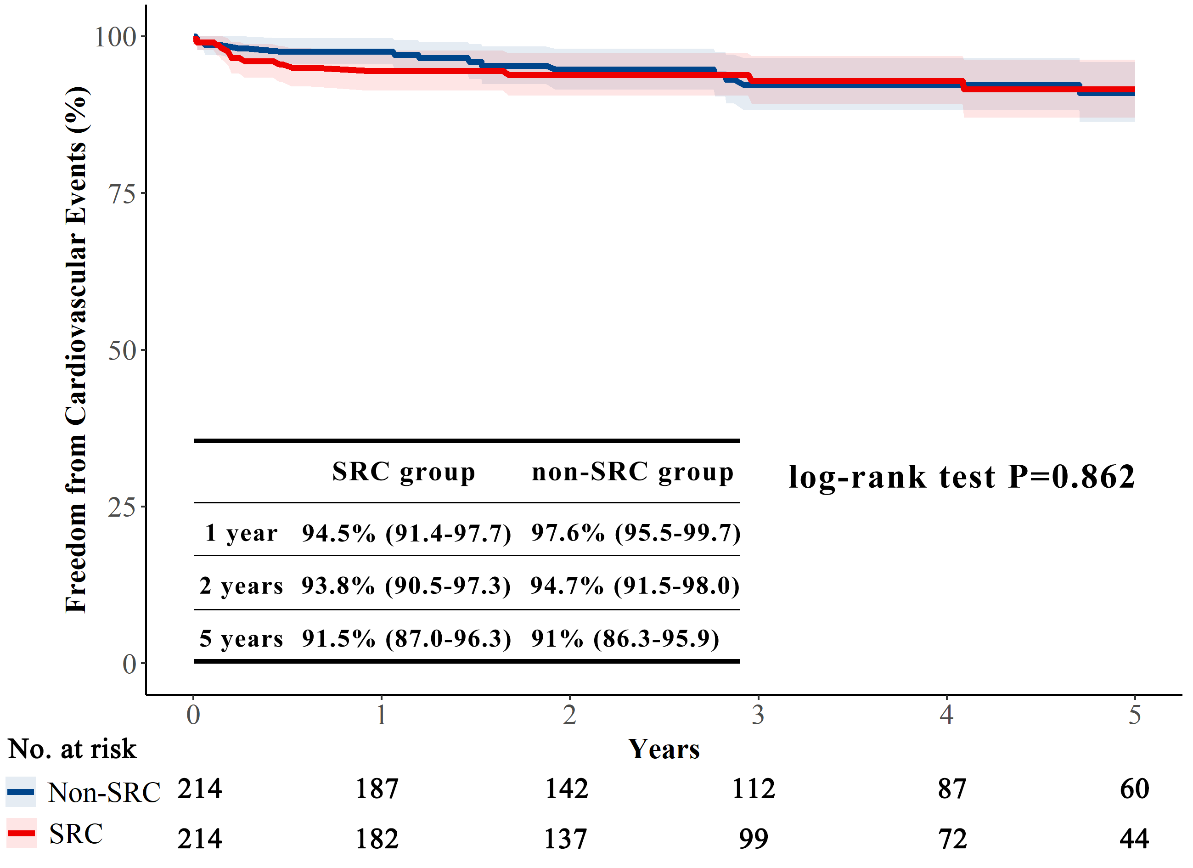


**Supplementary Fig. 6. Kaplan-Meier survival analysis of cardiovascular events in the matched population.** The difference between the SRC and non-SRC groups was assessed with the log-rank test. Freedom from cardiovascular events in the SRC group was similar to that of the non-SRC group (*p* = 0.862). SRC, simple renal cysts.
