# Supplementary material for: Long-Term Outcomes of Endovascular Treatment for Type B Aortic Dissection with Simple Renal Cysts: A Multicenter Retrospective Study
Source: Rev Cardiovasc Med. 2022 Jun 24;23(7):226. doi: 10.31083/j.rcm2307226 (PMC11266766; doi:10.31083/j.rcm2307226)
Supplement: Supplementary file 1 [file 2153-8174-23-7-226-s1.zip › 2153-8174-23-7-226-s1/v1-Supplementary Tables.docx]

Supplementary Table 1. Clinical centers.

| **Institutions** | **Patients included** |
| --- | --- |
| The First Affiliated Hospital of the Navy Medical University, Shanghai, China | 427 |
| Jiangmen Central Hospital, Guangdong, China | 60 |
| The First Affiliated Hospital of Qingdao University, Qingdao, Shandong, China | 227 |

Supplementary Table 2. Short-term outcomes in the overall and matched population.

|  | **Overall** | | | | **Matched population** | | | |
| --- | --- | --- | --- | --- | --- | --- | --- | --- |
|  | **SRC group** | **Non-SRC group** | **SMD** | **P value** | **SRC group** | **Non-SRC group** | **SMD** | **P value** |
| **Variables** | **n=248** | **n=470** |  |  | **n=214** | **n=214** |  |  |
| ARAEs | 14 (5.7%) | 13 (2.8%) | 0.14 | 0.054 | 11 (5.1%) | 7 (3.3%) | 0.09 | 0.335 |
| Aortic-related mortality | 9 (3.6%) | 4 (0.9%) | 0.19 | 0.008 | 8 (3.7%) | 3 (1.4%) | 0.15 | 0.127 |
| Type I endoleak | 3 (1.2%) | 4 (0.9%) | 0.04 | 0.642 | 3 (1.4%) | 1 (0.5%) | 0.1 | 0.615 |
| New dissection | 1 (0.4%) | 1 (0.2%) | 0.03 | 0.645 | 0 | 0 | NA | NA |
| Retrograde AD | 1 (0.4%) | 3 (0.6%) | 0.03 | 0.687 | 0 | 2 (0.9%) | 0.14 | 0.499 |
| Rupture | 8 (3.2%) | 2 (0.4%) | 0.21 | 0.002 | 7 (3.3%) | 1 (0.5%) | 0.21 | 0.032 |
| Organ failure | 1 (0.4%) | 2 (0.4%) | 0 | 0.965 | 1 (0.5%) | 2 (0.9%) | 0.06 | 0.562 |
| Cardiovascular events | 2 (0.8%) | 3 (0.6%) | 0.02 | 0.797 | 2 (0.9%) | 3 (1.4%) | 0.14 | 0.653 |
| All-cause mortality | 10 (4.0%) | 6 (1.3%) | 0.17 | 0.017 | 9 (4.2%) | 4 (1.9%) | 0.14 | 0.159 |

Values are reported as n (%) or median (interquartile range). SRC, simple renal cyst; ARAEs, aortic-related adverse events; NA, not available; AD, aortic dissection; SMD, standardized mean difference.

Supplementary Table 3. Long-term outcomes in the total population.

|  | **SRC group** | | **Non-SRC group** | | **P value** |
| --- | --- | --- | --- | --- | --- |
| **Variables** | **n=248** | | **n=470** | |  |
| **Freedom from ARAEs** |  |  |  |  | 0.063 |
| 1 year | 86.90% | (82.7-81.2) | 91.30% | (88.8-93.9) |  |
| 2 years | 82.80% | (78.1-87.7) | 88.30% | (85.3-91.3) |  |
| 5 years | 77.70% | (72.1-83.8) | 81.80% | (77.8-86.0) |  |
| **Freedom from all-cause mortality** |  |  |  |  | 0.054 |
| 1 year | 90.70% | (87.2-94.4) | 96.80% | (95.2-98.4) |  |
| 2 years | 90.30% | (86.6-94.1) | 95.30% | (93.3-97.3) |  |
| 5 years | 87.10% | (82.7-91.7) | 89.90% | (86.8-93.2) |  |
| **Freedom from cardiovascular events** |  |  |  |  | 0.414 |
| 1 year | 95.20% | (92.5-98.0) | 98.50% | (97.3-99.6) |  |
| 2 years | 94.20% | (91.2-97.3) | 96.30% | (94.5-98.2) |  |
| 5 years | 92.10% | (88.0-96.4) | 92.90% | (90.1-95.8) |  |

Kaplan–Meier estimates for aortic-related mortality, all-cause mortality and cardiovascular events. SRC, simple renal cyst; ARAEs, aortic-related adverse events.

Supplementary Table 4. Univariable Cox hazard analysis of ARAEs in the matched population.

| **Variables** | **Hazard ratio (95% CI)** | **P value** |
| --- | --- | --- |
| **SRC** | **1.80 (1.17, 2.80)** | **0.008** |
| Age | 1.00 (0.98, 1.02) | 0.893 |
| Male | 1.22 (0.69, 2.17) | 0.493 |
| BMI | 1.00 (0.94, 1.05) | 0.869 |
| Smoking | 0.72 (0.47, 1.10) | 0.123 |
| Drinking | 0.83 (0.49, 1.41) | 0.496 |
| Hypertension | 1.73 (0.96, 3.13) | 0.068 |
| CAD | 0.97 (0.39, 2.39) | 0.944 |
| Arrhythmia | 0.65 (0.31, 1.34) | 0.24 |
| **Stroke** | **2.97 (1.57, 5.60)** | **0.001** |
| **COPD** | **0.40 (0.15, 1.08)** | **0.072** |
| Diabetes mellitus | 0.88 (0.41, 1.91) | 0.753 |
| CKD | 1.57 (0.68, 3.59) | 0.29 |
| SBP at admission | 1.00 (0.99, 1.01) | 0.914 |
| DBP at admission | 1.00 (0.98, 1.02) | 0.878 |
| Pleural effusion | 1.26 (0.82, 1.93) | 0.298 |
| **Malperfusion** | **2.16 (0.94, 4.96)** | **0.069** |
| Diameters of ascending aorta | 1.41 (0.76, 2.64) | 0.278 |
| LVEF | 1.02 (0.96, 1.09) | 0.511 |
| LVFS | 1.03 (0.94, 1.12) | 0.562 |
| ACEI/ARB | 1.25 (0.82, 1.92) | 0.304 |
| α-blocker | 0.90 (0.58, 1.39) | 0.628 |
| β-blocker | 0.87 (0.56, 1.37) | 0.561 |
| CCB | 1.06 (0.66, 1.71) | 0.804 |
| diuretic | 1.28 (0.72, 2.28) | 0.391 |
| Arch type |  |  |
| I | Reference |  |
| II | 1.42 (0.67, 3.00) | 0.359 |
| III | 1.39 (0.85, 2.28) | 0.196 |
| Diameters of maximum ascending aorta | 1.03 (0.97, 1.09) | 0.358 |
| **Diameters of maximum descending aorta** | **1.02 (1.00, 1.04)** | **0.025** |
| Calcification |  |  |
| None | Reference |  |
| Mild | 0.91 (0.56, 1.48) | 0.714 |
| Moderate | 1.38 (0.74, 2.58) | 0.307 |
| Severe | 1.60 (0.57, 4.46) | 0.369 |
| Thrombosis |  |  |
| Patent | Reference |  |
| Partial | 1.18 (0.73, 1.90) | 0.503 |
| Complete | 0.95 (0.48, 1.87) | 0.872 |
| ULP | 1.39 (0.64, 3.00) | 0.404 |
| **Operation time** | **1.00 (1.00, 1.01)** | **0.075** |
| Oversize | 0.94 (0.86, 1.02) | 0.126 |
| Length of proximal landing zone | 1.01 (0.98, 1.03) | 0.566 |
| Timing of operation |  |  |
| Acute phase | Reference |  |
| Subacute phase | 1.33 (0.82, 2.14) | 0.247 |
| **Chronic phase** | **2.28 (1.30, 3.99)** | **0.004** |
| Hybrid approach | 2.97 (0.73, 12.07) | 0.129 |
| **Chimney technique** | **1.81 (1.13, 2.88)** | **0.013** |
| Adjunctive procedure | 1.05 (0.62, 1.76) | 0.861 |
| Era | 1.01 (0.66, 1.55) | 0.968 |

ARAEs, aortic-related adverse events; CI, confidence interval; SRC, simple renal cyst; BMI, body mass index; CAD, coronary artery disease; COPD, chronic occlusive pulmonary disease; CKD, chronic kidney disease; SBP, systolic blood pressure; DBP, diastolic blood pressure; LVEF, left ventricular ejection fraction; LVFS, left ventricular fraction shortening; ACEI/ARB, angiotensin-converting enzyme inhibitor/angiotensin receptor blocker; ULP, ulcer-like projection.

Supplementary Table 5. Subgroup analyses of ARAEs in the matched population.

| **Subgroup** | **Hazard ratio (95%CI)** | **P value** | **P for interaction*** |
| --- | --- | --- | --- |
| **Age group** |  |  | 0.429 |
| <60 | 2.05 (1.08-3.87) | 0.027 |  |
| ≥60 | 2.15 (1.15-4.03) | 0.017 |  |
| **Stroke** |  |  | 0.945 |
| no | 1.82 (1.15, 2.88) | 0.01 |  |
| yes | 4.61 (1.61, 13.19) | 0.004 |  |
| **Malperfusion** |  |  | 0.399 |
| no | 1.83 (1.16-2.91) | 0.01 |  |
| yes | 3.63 (1.51-8.76) | 0.004 |  |
| **Timing of operation** |  |  | 0.963 |
| Acute phase | 1.97 (0.99-3.91) | 0.052 |  |
| Subacute phase | 2.75 (1.31-5.75) | 0.007 |  |
| Chronic phase | 4.34 (1.91-9.84) | <0.0001 |  |
| **Chimney procedure** |  |  | 0.25 |
| no | 1.51 (0.91-2.51) | 0.111 |  |
| yes | 3.18 (1.76-5.75) | <0.0001 |  |
| **Adjunctive procedure** |  |  | 0.524 |
| no | 1.68 (1.03-2.75) | 0.037 |  |
| yes | 2.05 (1.03-4.05) | 0.04 |  |
| **Hybrid approach** |  |  | 0.321 |
| no | 1.84 (1.18, 2.86) | 0.007 |  |
| yes | 20.11 (2.68, 150.83) | 0.031 |  |

*Interaction between SRC and hypertension on ARAEs were investigated. ARAEs, aortic-related adverse events; SRC, simple renal cyst; CI, confidence interval.
